# Towards a better understanding of NHS secondary and social care backlogs: qualitative perspectives on waiting lists, deferrals and delays by disabled people from minoritised ethnic groups

**DOI:** 10.1136/bmjopen-2024-091182

**Published:** 2025-04-14

**Authors:** Carol Rivas

**Affiliations:** 1 Social Research Institute, UCL, London, UK

**Keywords:** COVID-19, Health Services, Bed Occupancy, Capacity Building, Disabled Persons, Waiting lists

## Abstract

**Abstract:**

**Objectives:**

To explore how backlogs in health and social care were perceived by and impacted disabled people from minoritised ethnic groups, with a view to improving their experiences and social, health and well-being outcomes.

**Design:**

Interview and workshop-based qualitative study as part of a larger mixed-methods study; main analysis is based specifically on the interviews.

**Setting:**

Primary and secondary care in the UK.

**Participants:**

271 participants aged 18+ with a chronic condition or impairment associated with disability, white British and from several minoritised ethnic groups, resident in the UK for at least 3 months of the pandemic, using quota sampling to ensure the recruitment of different disability-ethnicity combinations. Four ‘key informants’ or relevant others by virtue of their work (a medical general practitioner (GP), community leader, charity representative, member of parliament).

**Main outcome measures:**

Experiences of care backlogs.

**Results:**

Three distinct categories of care backlog left people suffering for months to years, worsening their condition and attitudes to the National Health Service. These were waiting lists for new patient secondary care (including bottlenecks in GP referrals), deferrals (in patient help-seeking or diagnostic appointments) and delays with existing care plans. Within each category, nuances in experiences, such as feelings of being in limbo when waits and delays were undefined, suggested subcategorisations that are helpful for determining future policy. Disability had more effect on experiences than ethnicity, though an intersection with waiting lists and referral models from other countries was reported.

**Conclusions:**

Different types of backlog require different government considerations. Work is needed to restore patient confidence and encourage help-seeking, as well as improving access to general practice, to encourage those people to use healthcare who ceased doing so during the pandemic. Referral processes may need substantial reform to remove GP bottlenecks. There should be more transparency about wait times and the way certain conditions are prioritised for patients on long waiting lists or with cancelled appointments. Neglected specialties such as gynaecology and those currently overloaded such as mental healthcare should receive particular support, and alternative services should be considered.

**Strengths and limitations of this study**

- We were inclusive, recruiting across a wide range of impairments (including undiagnosed impairments) and ethnic groups, making this the most in-depth study of the lived experiences of these groups, in the context of their health and social care.
- Community coresearchers were used to increase reach and include undocumented migrants and others with precarious status.
- A majority of interviews were online and thus may have excluded some older and more disabled participants, though a significant number of interviews were face-to-face, and there was also limited participation from the devolved nations since the study focus was on England.
- There were formal translators/interpreters in only two of the six sites, though consent was fully informed across all sites.
- The qualitative data used in this analysis include a greater proportion of South Asian people than other ethnic groups, though this was corrected for in analyses.

## Introduction

 In July 2024, there was a general election in the UK; a UK Prime Minister’s term of office lasts a maximum of 5 years. National Health Service (NHS) waiting times were a key consideration in the different party manifestos at this time, reflecting public concerns. For example, a 2024 YouGov poll^1^ reported greater dissatisfaction in the NHS than in 2020 and 2022. Moreover, 56% of respondents believed the NHS will deteriorate further, and only 14% that it will improve in the near future.^1^ These statistics, in comparing data across recent years, suggest standards of pandemic and postpandemic health and social care have led to public dissatisfaction. However, it is more accurate to say the pandemic made more apparent the troubles the NHS had been facing for some years prior. Chronic staff shortages had cut across the different sectors, exacerbated by many factors, including a 2012 increase in the cost to students of expensive 5-year UK medical school undergraduate degree fees, and a 2016 removal of bursaries for prospective nurses and midwives. Remaining staff were overworked, suffering from exhaustion and burnout,^2^ often doing overtime on ‘goodwill’,^3^ and there was a heavy use of agency or locum staff or staff recruited from overseas; staff retention became an issue.^4^,^6^ These factors contributed to long waiting times for diagnostic tests, emergency care, ambulance services^7^ and elective care, which rose consistently between 2012 and 2019^6^; in December 2019, 4.6 million people were on a waiting list.^8 9^ Across the sectors, government targets were often missed.^5 8 9^

In parallel, there was a policy to compress bed stays, with the aim of more efficient use of resources, in the context of reduced funding in real terms and service reorganisations as well as treatment advances.^10^ Other shifts in policy led to the care of people with mental illness and intellectual disabilities being moved from hospital to institutional settings, and an increase in local community care and support. New models of care centred on prevention, early intervention, reduced admissions and support for independent home living across health and social care.^11 12^ Responsibility for the long-term care of older people transferred from the NHS to social services, taking place at their own residential or nursing homes.^13^ These changes meant the government felt confident to reduce the number of physical NHS hospital beds in England from around 299 000 in 1987/1988 to 141 000 in 2019/2020.^10^ However, the population had expanded over this period, and austerity had compromised the health of the nation.^10^ At the start of the pandemic, in consequence, not only did the UK have fewer hospital beds per 1000 inhabitants than many other comparable health systems, these were already overstretched.^10^ For example, over 2019, an average of 90.2% of general and acute beds were occupied.^10^ Government capital funding had also failed to keep pace with maintenance requirements, inflation and clinical need, and many hospital and general practitioner (GP) buildings across the UK, and NHS equipment, had become unfit for purpose.^5 10^ Government policies had attempted to mitigate the issues by pushing for a move to more private care, but this was only affordable for some, and widened inequities in care.

Thus, when the pandemic began, chronic staffing, bed space, estates and equipment issues had already forced the NHS onto its knees. The pandemic itself led not only to additional demand on services, including acute and critical care, but also increased staff absences, with COVID-19 infection and isolation adding to the issues of sickness absence due to burnout and stress. Absences were exacerbated by an initial lack of personal protective equipment for staff, which led to a decision to not recommend this early in the pandemic for members of the public, and hence more sick patients requiring hospital care, in a vicious cycle. Lockdowns and other restrictions, as well as pandemic fears, led to an immediate reduction in international recruitment, and existing international staff often returned home to be with family.^5^ The immediate requirement was for more staff in critical and high-need services, which was achieved in the first months of the pandemic by redeploying staff, including asking allied healthcare professionals to assist in other specialty areas such as surgery, asking retired and non-practising staff to return to clinical practice, enabling clinical students to join the NHS staff force earlier than normal in their degrees, and deploying public volunteers (most evident in the pandemic vaccination centres). Usable hospital beds, that is, beds with sufficient equipment and the staff to manage them, lessened further because of reduced staffing due to sickness or redeployment to other areas of the hospital such as critical care.^4^ Social distancing and other infection-control measures also limited the bed numbers in a ward. Outdated equipment, including poor video-conferencing facilities for remote consultations, could not cope with the need. Non-COVID care was temporarily cancelled, excepting the most urgent cases, including some cancer treatments. Ambulance teams were overloaded, and their response times increased considerably, to a mean in December 2022 of over 1.5 hours for a category 2 call (eg, for suspected heart attacks and strokes),^7^ thus missing the ‘golden hour’ of intervention, and the government target of 18 min. Beds were urgently needed to cope with the record numbers of admissions due to COVID-19 in particular, but also, as the pandemic progressed, other conditions that worsened because of lack of available care and backlogs. The so-called new Nightingale wards, essentially ‘pop-ups’, generally lacked the staff to use them.^14 15^ Early discharge of patients into care or their own homes was a particular challenge when the patient lived in substandard accommodation, which was more likely with minoritised groups. Block-booking agreements with private sector hospitals became common.^2 16^

These various pandemic impacts all led to patients becoming first sympathetic to NHS staff efforts and then increasingly disgruntled with what was perceived as a lack of caring as well as care. It was in this context that I developed the CICADA-ME study (Coronavirus Chronic Conditions and Disabilities Awareness Study-Migrants and Ethnic groups; hereafter CICADA), which aimed to explore the pandemic experiences of people from minoritised ethnic groups who had impairments and chronic conditions associated with disability. This focus was important. Data had emerged that showed the pandemic was widening pre-existing inequities in health and social care access and use for these groups.^17^,^20^ A 2021 report had described deprivation as being a key marker of inequities in waiting lists specifically. Though it did not link this to ethnicity and disability, people from minoritised groups are more likely to be deprived because of structural discrimination.^21^ Therefore, my particular interest was in whether and how disability and ethnicity, along with citizenship status, intersected^22^ to compound inequities because of structural discrimination.

In this paper, I consider what participants said about the waiting lists and diagnostic delays that were subsequently a focus in the 2024 election party manifestos. The CICADA data remain topical. By the end of 2024, the NHS had still not returned to even overstretched prepandemic levels of care. Ambulance responses have only slightly improved.^7^ In January 2024, a daily average of 5600 patients waited longer than 12 hours in Accident and Emergency (A&E).^8^ The waiting list for hospital treatment following a GP referral reached a record of nearly 7.8 million in September 2023.^8 9^ While England’s waiting lists began to fall in 2024, the Institute for Fiscal Studies (IFS) has predicted, from current trends, that it will take years to reach pre-COVID-19 pandemic levels.^8^ In January 2025, the new UK Prime Minister, Keir Starmer, introduced plans to speed up this recovery.^23^ Patients will be able to get a direct referral from their GP for diagnostic and other tests and scans, replacing the previous requirement for patients to see both a GP and then a consultant first, and with many being offered a follow-up consultation on the same day as their scans or tests. Routine surgery such as hip and knee replacements will also be protected from cancellations and delays caused by seasonal or unexpected increased demands on the NHS more generally. However, there was no mention in these plans of ways to address inequitable service provisions. In 2022, the Nuffield Trust had reported that elective care had decreased disproportionately for Asian people in the UK.^24^ In October 2024, they found that people from the most deprived areas are much more likely to still be on waiting lists for elective care, that young black people experience longer waits in A&E, and that there are particularly amplified waiting lists for gynaecological care.^25^ They have called for more research to explore this. The CICADA study goes some way to address this need.

## Methods

### The CICADA study

The CICADA study was a mixed-methods, strengths and assets-based study, which adhered to embodiment disability models^26 27^ and intersectionality theory.^22^ The study included secondary data analysis, but its focus was on primary data collection, with a new survey in 2021 involving 4326 complete responders who were invited to repeat the survey two further times in 2022, and qualitative data collection and analysis. This paper reports on interview data from the qualitative data stream, in which the backlog in care featured strongly in participants’ stories. Full details of the methods are reported elsewhere.^28 29^ Here, I summarise these.

### The qualitative work

The focus in the qualitative work was on adults aged 18+ of Arab (Middle Eastern and North African), South Asian, African, Central/East European and white British heritage with and without disability. Purposive quota sampling was used to capture all possible combinations of the study’s six ‘disability’, one non-disabled and five ethnicity categories (table 1). Within these, a range of citizenship states was sought (undocumented, on temporary visas, with indefinite leave to remain, with British citizenship). Any incurable condition or impairment that affected daily living was included and matched to six categories, adapted from UK Government Statistical Service harmonised data recommendations on disability, with two additions as recommended by the advisory group (to represent cancer and brain hyperexcitability (epilepsy or migraines)). Self-diagnoses were included to capture conditions that typically take years to be diagnosed.

**Table 1:** Quota sampling combinations, initially aiming for 5 per cell and then 7 once top-up funds were received in spring 2021

Condition—to matchUK administrative data disability categoriesEthnicity	People born outside the UK from parents not born in the UK					
	Arab	Sub-Saharan African	Central/East European	South Asian	Born in the UK from parents not born in the UK	Native White British
Mental health						
Mobility						
Stamina/breathing/ fatigue (including heart)						
Sensorial						
Cognitive						
Food-relevant						
No condition/disability						

The six recruitment sites, including a mix of local communities well served by immigrant-specific services and less service-rich communities, were all in England and comprised Manchester and the Northwest coast, Yorkshire, London, South East England, Newcastle and Cumbria, and the Midlands.

The original target for the qualitative work was 210 semistructured interviews, 2 workshops with interviewees 5 and 10 months later and up to 15 key informant (relevant professional) interviews. Mixed stakeholder cocreate workshops to develop rapid-impact solutions to issues, also undertaken, are reported in a separate paper. Participants were recruited using posters, adverts, snowballing and invitations sent out via the study’s various networks, partners, community coresearchers and clinical research networks. Convenience sampling and inclusive principles meant anyone who responded to these by showing an interest in taking part did so unless screening talks preinterview made it clear they did not fit the inclusion criteria; only six were gently told they were not eligible. One-third of interviews were undertaken face-to-face and 30 were conducted by telephone. One was by email because the participant had difficulty talking due to an intellectual disability. The remainder were completed using Teams or Zoom. All participants received £20 per interview.

Separate face-to-face or online workshops (none hybrid) or, if preferred, repeat interviews were offered in May and September 2022 for interviewees who had provided contact details and completed interviews between 1 July 2021 and 20 October 2021. Following top-up funding in spring 2022 the target increased to 280 interviews, with recruitment and interviews continuing from 1 May 2022 to 15 September 2022. Recruitment stopped at 274 due to the end of the study. Two interviews could not be used because the digital consent forms were accidentally deleted, and one because the interview was accidentally deleted before transcription. All remaining 271 first interviews are included in analyses, and longitudinal components are described (ie, the later interview data were compared with earlier data as part of the analysis, with differences reported in this paper). Workshops and repeat interviews explored the team’s interpretation of interview themes (ensuring credibility) and also subsequent change, assets and strengths, issues and potential solutions. All interviews and workshops were structured using topic guides. These explored the different aspects of daily living during the study period, but the main focus was on health and social care experiences; we began with open questions followed by probes. The workshops also used illustrative vignettes recorded by the study’s patient and public advisory group (PAG) using verbatim data. Interviewers and facilitators made field notes and summaries; this helped the field researchers to gel as a team and reflect together on their cultural understandings and cultural humility.^30^ It also aided quota sampling checks. Participants were offered £40 for the May 2022 workshops, while for the September 2022 workshops, £40 was offered for face-to-face participation and £20 for online (including two who chose repeat interviews), since pandemic restrictions had mostly ended. Vignettes were updated in September to incorporate May workshop data and shortened, and design thinking tools added for structure: patient care journey maps; future planning vision cones and structured brainstorming.

Particular attention was paid to anonymisation and confidentiality as some participants were undocumented; some community researchers, therefore, only provided transcripts, with all identifying data deleted immediately after they produced these. Otherwise, raw data were stored on a secure UCL Data Safe Haven until data cleaning had been completed and were then deleted.

### Patient and public involvement and the qualitative work

The study was inherently participatory. Members of the public and patients were involved from the grant application writing stage and throughout, with two as coapplicants who helped shape the research questions and design. Study materials were piloted within relevant communities. PAG members were trained at the start of the study to become coresearchers through all study stages except for interviewing. 11 community coresearchers local to the recruitment sites were also trained, who were economic migrants and asylum seekers often with chronic conditions themselves. Recruitment and interviews were undertaken by the core team at University College London (both female, one of whom was an Indian migrant, one white British, both with experience in similar research), by the study’s main community partners (Born in Bradford and Bromley by Bow Community Centre) and by eight of the trained community coresearchers. Completed coresearch work was paid according to the funder guidance. Some PAG members and coresearchers contributed as coauthors to two papers drafted by the central team; they also helped develop a public theatre show as well as intervention recommendations in codesign workshops. Several coresearchers helped with data analysis, and two became involved in later stages of the work, including writing the first drafts of reports and papers. PAG members, with one of the community coresearchers, helped facilitate workshop discussions and ensured workshops were accessible and inclusive, which included their audiovisual recording of vignettes. Considerations of research burden by the PAG were especially important for the workshops, leading to the second series being curtailed in September 2022. The study lead (the author) also had relevant lived experience.

### Qualitative analysis

The main qualitative analysis as reported here used the Framework approach^31^ with data management in NVivo V.12 (QSR/Lumivero), subsequently moved into Microsoft Excel. Deductively determined themes (the broad topics that shaped topic guides: Intersectionalities; Behavioural responses to the pandemic; Access to resources, support, health and social care; Social networks; Mental and physical well-being and quality of life; Coping; Local and regional differences; Future policy suggestions) were augmented by inductive themes (online supplemental files 1; 2). Workshop validation and an inter-rater reliability exercise between three core team members involving coding and discussion until values of 75%+ were achieved for the key themes, and the study’s participatory work more generally, ensured credibility. Transferability was ensured with full description, data triangulation and sensitivity analyses, confirmability using illustrative extracts and dependability via transparent methods and data archiving. Workshop data have not been directly used in this paper, but discussions within these were used to confirm interpretations.

## Results

Interviews lasted 25–90 min, workshops 2 hours. Altogether 80 of the total 271 interviews were undertaken by partners and community coresearchers. No-one dropped out. 10 were not held in English and were translated by the community coresearchers who undertook them. There were no quality checks on the translations as the original data were immediately deleted at the request of the interviewees, some of whom were undocumented. For the follow-on research workshops in May and September 2022, 134 were invited by email; 104 attended in May with three online workshops and two face-to-face (in London and Bradford). In September 2022, we held two London face-to-face sessions (n=22), two small online sessions with participants from the Midlands (n=11)—converted from a local face-to-face session at their request—and two individual remote interviews. Each workshop was capped at 30 and used breakout groups or tables. Reasons for non-attendance in September were mostly that people had returned to their jobs and normal lives and did not have the same availability.

Detailed demographics of the interviews are shown in table 2. Despite preinterview screening, some ethnic group identities and sites fell outside the initial sampling criteria. In analysis, these were included because fuzzy boundaries and decategorising are in keeping with an intersectional approach, and moreover, it would be unethical in terms of participant burden to collect data that were not used. However, these additional data were compared with the core data (ie, those data satisfying the initial sampling criteria) in a form of sensitivity analysis to see if the initial sampling decisions were critical. Where they were, the differences are reported.

**Table 2:** Public participant demographics (n=271)*

Category	Subcategory	Proportion of total 271
Ethnicity	South Asian	34.3%
	African	11.1%
	Central/East European	10%
	Arab	26.2%
	Undocumented	3.7%
	White—British, Irish	7.4%
	Other	7.3%
Age	18–24	12.3%
	25–34	42.3%
	35–44	24.5%
	45–54	12.6%
	55–64	5.9%
	65+	2.4%
Gender	Male	46.4%
	Female	52.5%
	Gender non-conforming	0.4%
Site	Southeast England	7.8%
	London	40.2%
	Midlands	11.1%
	Manchester and NW Coast	13.3%
	Yorkshire	10.3%
	Cumbria and Newcastle area	6.3%
	Scotland, Wales	8.5%
Condition/ impairment†	Mental health	24.7%
	Mobility	31.0%
	Dexterity	1.1%
	Stamina (breathing/ fatigue and cardiovascular issues—as per government harmonised data)	36.2%
	Sensorial (a fifth being deaf, the remainder blind)	5.9%
	Neurodivergent	7.4%
	Cognitive	1.5%
	Food-relevant	17.3%
	Brain hyperexcitability	6.6%
	Cancer	6.3%
	Non-disabled (ie, no condition/impairment)(across ethnic groups)	7.4%
	Multimorbidities	33.6%

* NB. Note that uUndocumented figures are a minimum as some participants did not wish us to know their citizenship status. This also means we have not written identifying details for undocumented migrants in the text and in some cases, we have not disclosed their undocumented status for disaggregated data.

† aAll incidents, used for quota sampling; figures will add up to more than 100% given that 33.6% of participants had more than one condition.

The team was successful in recruiting for all combinations in table 1, except for African or Central/East European participants with sensorial loss. The samples were broadly representative of national data.^32 33^ There was a spread of ages, though with more participants below 55 years which may partly reflect a heavy reliance on the internet for interviewing during pandemic restrictions. Females slightly outnumbered males. Given that the index condition identified at screening did not necessarily dominate the participant’s life if they had comorbidities, and given particular clusters of comorbidities, in analysis a multimorbidity approach was used, with holistic consideration of the experiences of someone with multiple conditions.^34^ Multiple comorbidities were proportionately most common among South Asian participants in the data, indicating complementarity with national health records data.^35^ Only four key informants were recruited before the end of the study, due to UK political instabilities at the time of their recruitment. These were a GP, a community leader, a member of parliament and a charity representative.

### Framework analysis

**Figure 1:**
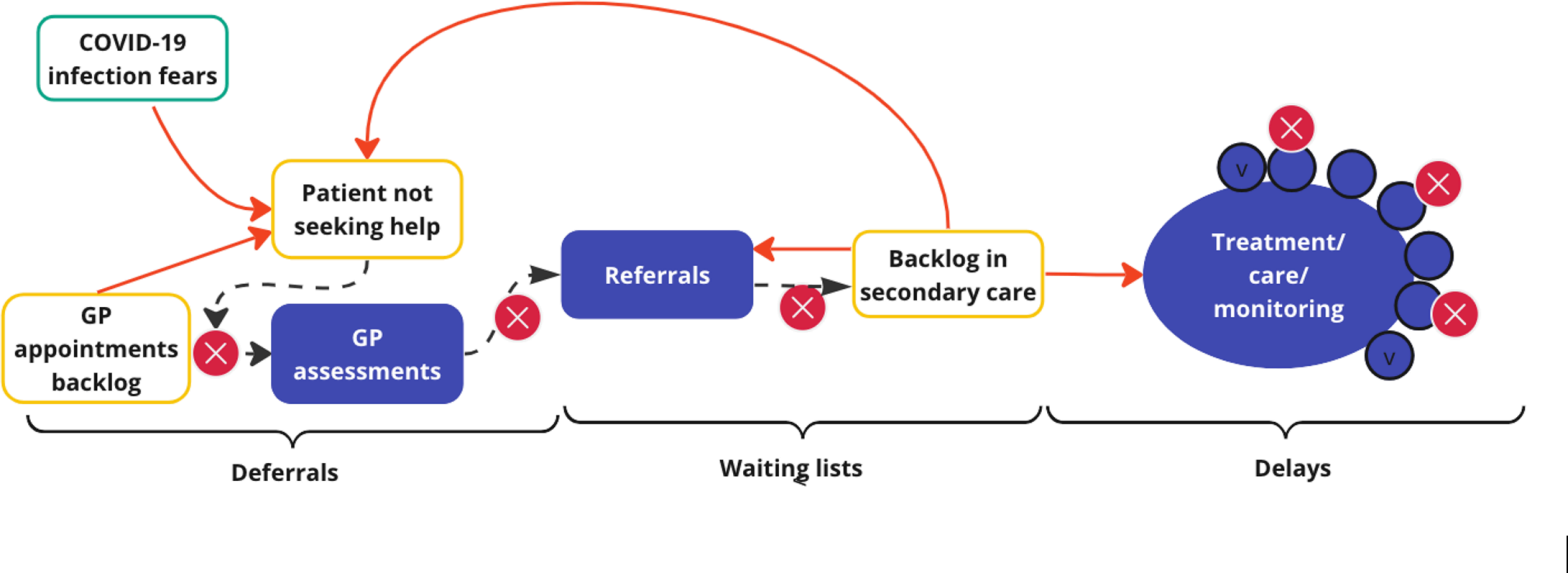
Processes impacting on health and social care service delivery. Solid (red) arrows are negative feedback loops. Filled (blue) shapes are the usual pathway to care. Unfilled shapes are issues in the normal process. X (red) shapes and dashed arrows show where these disrupt the process, leading to delays and cancellations. GP, general practitioner.

The framework analysis showed many participants bewailed the backlogs in secondary and social care. While data from all 271 were considered for context, 73 participants provided detail on backlogs; exploration of these data revealed the usefulness of subdividing them into ‘deferrals’, ‘waiting lists’ and ‘delays’ to best reflect what participants said (table 3, figure 1). Table 3 shows how this adds to previous considerations by the British Medical Association (BMA)^36^ and the IFS and helps to explain some of the questions raised by the IFS in June 2024.^37^ On the basis of the data, I have defined deferrals as occurring when patients, knowing about extended waiting lists, or unable to book a GP appointment, or fearing COVID-19, put off help-seeking, or where primary care diagnostic tests and reviews or discussion of their results are held back by healthcare providers. I define waiting lists as lists joined when a person is newly booked into secondary or social care for a diagnosis or procedure or support following a successful GP referral (the definition used in the NHS benchmarking for the elective care waiting list/‘RTT’ (referral to treatment) waiting list), or where GP referrals have been delayed, cancelled or refused due to a lack of capacity in primary, secondary or social care, in other words where there has been an ‘active’ block on a patient joining the waiting lists. I define delays as occurring when existing treatment or monitoring plans or social care visits are less frequent or temporarily abandoned or cancelled.

**Table 3:** Different categories of backlog in care found in CICADA study data

	Described by the BMA^32^	Described by the IFS^33^	Arising from the CICADA study data
**Deferrals**			
Patients who have not yet seen their GP about symptoms that would ordinarily lead to a referral, due to concerns of burdening the health service, issues making an appointment or fears around COVID-19 infection.	Not quite	?Proposed as a supposition	Yes
Patients for whom diagnostic tests or assessments, or non-routine assessments or discussion of the results of these to enable a diagnosis, have been cancelled, postponed or delayed.	No	Yes (some patients also on the elective waiting list)	Yes
**Waiting lists**			
New patients on a secondary care treatment/social care waiting list who would have expected to have been seen already were it not for the pandemic (elective care waiting list)	Yes	Yes	Yes
Patients who had GP referrals delayed, cancelled or refused due to a lack of capacity in primary, secondary or social care^3^ (waiting for referrals)	Yes (separates out refusals from delays/cancellations)	Notes a fall in referrals but calls this a ‘puzzle’	Yes
**Delays**			
Patients who have already had a secondary care consultation but have had planned management or interventions or support cancelled with no suggestion that they will be provided in the future, thus apparently removed from active lists.	Combined, but CICADA data show specific differences.	No	Yes
Patients who have already had a secondary care consultation but have had management or interventions or support delayed or postponed (but with the patient being given the expectation they will happen quite soon)		No	Yes
Patients whose regular management or monitoring or review consultations or social care meetings were less frequent but continued.	no	No	Yes

BMA, British Medical Association; CICADA, Coronavirus Chronic Conditions and Disabilities Awareness; IFS, Institute for Fiscal Studies.

All three backlog subtypes left people suffering for months to years, often in chronic pain or with mental ill health, without diagnoses to enable support, without therapy, and without rehabilitation and enablement measures such as home equipment. Consequently, their condition and expectations of the NHS worsened. I discuss waiting lists before deferrals below because deferrals were a consequence of the waiting lists. I then consider the strategies participants used to get seen in the face of these care backlogs.

### Elective care waiting lists (waiting lists type A)

Extended waiting lists for new patients in secondary healthcare and social care were treated pragmatically by some participants as inevitable with the pandemic, though they were not happy about the consequences. Social care waiting list complaints centred on the lack of a dedicated formal carer or unsuitable housing.

Healthcare waiting times mostly exceeded 6 months; participants believed critical conditions were prioritised with the longest specified times being for endometriosis (1 year) and talking therapies (up to 2 years). The NHS Constitution target maximum wait for consultant-led elective treatment is 18 weeks from GP referral.^36^ Waiting times were also particularly exacerbated for those who needed interpreters:

Getting an interpreter was, is quite difficult. So for the normal hospital […] [don’t] need it because they don’t speak much. The problem is the mental health support if it’s on the phone or not on the phone. […] So they book in advance but that takes, there are waiting lists for that, so, it just makes everything longer. For GP don’t really need an interpreter because it’s really simple. (P236, South Asian, Food relevant/Mobility/Mental Health)

Participants usually described the wait as absolute, that is, patients were not offered interim care, nor given indications of waiting times, which was especially problematic for mental health issues and neurodivergent participants (who typically become extremely anxious when there is uncertainty). Participants felt treated dispassionately, forgotten and neglected. Many felt in limbo (a word often explicitly used), particularly in the later interviews.

Right now I'm in limbo, nothing has really been done to be honest to help me […] I called them up last week and they said 'You are on the waiting list' and […] they said 'We can't give you an estimate, the list is very long but you are on the waiting list'. […] So another six weeks later, I made another appointment with the GP saying my condition […] was getting worse but I haven't heard anything back from the pain clinic […] So she gave me their number and I called them directly. They said 'You are on the waiting list but we don't know how long it’s going to take'. […] It’s like talking to a robot on the other end of the phone. I don't blame them because they're just doing their job, they're just telling you the facts but nobody knows what you're going through day in and day out so you just need a bit of compassion or a bit of sympathy to say, 'You're on a long waiting list but there’s something else that we can do for you'. (P205, South Asian, Stamina/Respiratory)

A similar situation occurred for social, council and formal community care. Since these often involve more complex processes than other care, with multidisciplinary teams and private sector provision, sometimes professional networks or processes could break down, exacerbating feelings of neglect.

### Waiting for referrals (waiting lists type B)

Several participants said they had GP referrals delayed, cancelled or refused due to a lack of capacity in primary, secondary or social care. Most commonly, this was stated for mental healthcare, emanating from a crisis in the specialty caused by the pandemic: *They said […]they don't have enough space for mental support because so many people are coming to the doctors about it. (P43, South Asian, Respiratory/Mental Health*). This crisis had occurred because many adults—participants and the general population—experienced new mental health issues or exacerbations of existing ones, including suicidality in the pandemic.^37^,^39^ This was attributed by participants to loneliness, social isolation and stress from remote work, job loss, closed schools, stay-at-home orders, closed businesses, physical distancing, adversity experiences and infection fear. Deterioration in other conditions because of poor healthcare access was also linked to worsened mental health among participants.

Central and East European participants stated they were used to different systems in their countries of origin that bypassed the GP, speeding up the process and making it more likely a specialist would be seen. This is common in many other countries outside the UK.

For most of the things that should be available literally just by yourself, you need to actually go to your GP and be like, 'Hey, can you refer me to this?' I don't want to go to my GP every issue that I have. […] It’s also the fact that everything takes forever. If I am having, say if I want to get in touch with someone about my depressive episodes, I need to go to my GP, get them to actually have a conversation with me, for them to decide whether or not I actually need that help […] The fact is, GPs should not be the ones to decide whether or not you need outside mental health support […] I get that the healthcare system here works different, but it’s really not good. Especially for mental health, it’s really not good. (P276, Central/East European, Cognitive/Mental health)

### Patients waiting to seek help (deferrals type A)

Some participants had stopped help-seeking except for crisis care (“u*nless it is very very very crucial, I just keep quiet and sit at home*.” P12, South Asian, Sensorial), because of waiting lists and difficulties getting an appointment with a GP, and therefore, a referral, in the first place. This was most frequently mentioned for mental healthcare.

Going back to the GPs, now because of the Covid they take ages to do a referral, more than six weeks […] I've been going through psychology for a while and then that stopped […] So because the GP has to refer me every time I've given up. (P208, South Asian, epilepsy)

### Issues getting diagnoses (deferrals type B)

Participants reported long-deferred primary care diagnostic tests and non-routine assessments. This means conditions or their progression being confirmed at a later and more serious stage, resulting in a worse prognosis and unnecessary suffering for patients. It might also mean intermittent or relapsing-remitting conditions were not picked up, resulting in dismissed needs and diagnoses not being made.

And then when I was feeling really washed out and everything a few weeks ago, my GP asked me to do a blood test. The blood test isn't until the 10th of November [weeks after the interview] and it’s like it’s not going to be a true reflection of how I was feeling at the time because I was just completely wiped out. And I don't know how I'm going to feel in November when they take my blood test. (P205, South Asian, Stamina/Respiratory)

As the IFS has noted^37^ (table 3), some of these patients might also be on the elective care waiting list, for example, for MRI scans or ultrasound to confirm tentative GP diagnoses. People waiting for diagnoses, as with patients on the elective care waiting list, were more likely than other deferral patients to talk about being in limbo. This might occur, for example, when they had an indication from the GP that they might be eligible for the government clinically extremely vulnerable list but were excluded from it until their diagnosis was confirmed. This had considerable implications for their safety from COVID-19 infection and their care. The charity key informant told us that “*every GP practice has a call and recall team who spend their whole lives literally phoning up thousands of patients with long-term conditions and then triaging”* which they thought might reduce patients’ negative feelings. However, this was only mentioned by a couple of participants.

### Indefinite cessation of secondary care (delays type A)

Participants had planned management, interventions or support indefinitely cancelled for gender-reassignment therapy, podiatry and rehabilitation physiotherapy. They were given no expectation of their resumption, though the study data suggest this began to happen in 2022, so that I categorise them as a type of delay. In the meantime, inevitably, participant health suffered; many spoke of prolonged pain. As with long waiting lists, and patients waiting for a diagnosis, these indeterminate delays led people to enter a liminal space, lower their expectations, and even feel hopeless and suicidal:

The appointments have just come to a stop. […] So, I did feel sometimes that I’d be better off just killing myself to put an end to all of this. (P161, South Asian, Mobility)

### Revised secondary care appointments (delays type B)

There was a clear distinction in the data between existing patients with cancellations with no clear new date (delays type A), and existing patients given a revised date, who were initially less likely to feel in limbo, which I have grouped under delays type B. But revised dates in the near future could be broken, leading to an anxiety-provoking helter-skelter of hopes and disappointments.

They told me that they would put me on the waiting list for CBT therapy. […] they send me message that I will get my therapy in like […] three month’s time so I was like oh yes, wow, what happened. This is great […] then after these three months, […] they sent me like all your therapy will start in four weeks. And then two months later they were sending another text saying it was starting in four weeks. And being a person suffering from anxiety, I would really prefer if they told me it was starting six month’s time […] not like I think, okay, we’ll start soon, I’ll get help soon and then no I didn’t […] So it gives me more anxiety. (P185, Central/East European, Mental health)

The data suggest that only critical needs fell into this category. However, as with elective waiting lists, what was deemed critical and therefore prioritised was unclear. For example, while one participant with cancer got new appointments fairly quickly (delay type B), another said their kidney dialysis ceased (delay type A). Similarly, what constituted ‘critical’ for mental healthcare needs was also apparently open to interpretation:

And if you are suicidal you're not about to tell anyone. That comes out during counselling. So the crisis team were talking to me about whether I was suicidal […] And I know that there was a panic within me that was just on the horizon. But because I wasn't actually then I didn't get the immediate help. (P220, White British, Mental health)

And the doctor decided that because […] our family […] we’re supportive, he feels like [my sister] can't be put on an emergency list so they put her on a long waiting list […] she had mentioned that she was feeling suicidal. […] She was self-harming which was kind of bad […] And then after, she did attempt. (P43, South Asian)

Occasionally, type B cancellations were last-minute decisions because the clinician had COVID-19, which were described as poorly done, with the inconvenience pushed onto the patient, particularly upsetting for those with mental health or fatigue issues. One patient was even told to repeat the referral process, thus being removed from any lists, because cancellation by the hospital made the prior test results out-of-date.

I got an appointment and then they cancelled it the day before, and then I have to rebook it and I have to wait for hours on the phone to try to rebook it and then the phone goes off, things like that, it’s been really frustrating. (P16, South Asian, Stamina/Mental Health)

For me to go out of the house, I work at this thing with my doctor. We plan it, we organise it, we pace it. So that’s what I do, I plan, organise, pace my week. I’ve already now trained my brain three, four days earlier saying, I’m going to do this doctor’s appointment. It’s going to be okay, I’m not going to exert myself, I’m not going to overdo it, I’m going to take it easy because I need to save that energy for the next two days’ time. I need to now get ready and get out my house and go to an appointment. (P234, South Asian, Fatigue/Mobility)

Social care delays were typically contingent on the presence of particular staff, or staff social distancing or shielding (*I had a support worker coming in […] I think she’s been off sick herself. P159, African, multimorbidity*), whereas in healthcare, the care needs of COVID-19 patients also played a critical role.

### Long intervals between monitoring or review appointments (delays type C)

The third category of delay related to routine monitoring tests and reviews. Some cases represented a move from delay type A, but the long backlogs meant that in practice, participants felt no better off.

And for the past three months I've been trying to book an annual review. And they didn't have any clinics set up so they're still not inviting anybody in […] There’s got to be a backlog and they won't see me straightaway. They're saying it should be coming up soon […] But it’s been [18 months], it’s been a long time. (P206, South Asian, Multimorbidity)

Other participants had continued monitoring and review appointments through the pandemic, but they were less often, or face-to-face and remote monitoring appointments were alternated.

### Strategies to shorten waits

Participants were not necessarily passive in the face of the different backlogs in care. Some took control of their own health and social care when services did not meet their needs. A few found contact details and emailed hospital consultants, charities and social services themselves. Several said phrases such as “*You have to be very, very pushy to get your own appointment, because otherwise, nothing.” (P193, Central/East European*) or raised or threatened to raise complaints (*Because I think when they hear liability and things like that, they start paying attention, P222, South Asian*). People used struggle and fight metaphors that illustrated the drain on them emotionally or the negative stereotyping by healthcare staff.

I felt that every part of getting help was a struggle yes. And I had to ask many times again and again for help, and yes, it added up to my depression and hopelessness and anxiety. (P185, Central/East European, Mental health)

I had to fight really hard the last six months with their service that provides a nurse […] They stereotype us. I’m not the norm Indian woman. An Indian woman doesn’t make complaints. An Indian woman takes whatever the GP says and doesn't challenge them […] They put a label on me, I’m too challenging, I’m too neurotic. (P64, S Asian, Multimorbidity)

Empowerment sometimes involved playing the system rather than complaining, for example, some bypassed a stage in the process of getting to secondary care: *My wife came with an idea that we should go [straight] to the counsellor at the nearest hospital [for stress]. (P260, Arab, Mental health*)

Some white British and Central/East European participants felt empowered to obtain stop-gap care, particularly those seeking psychological or psychiatric support. For example, one used church-based counselling till she received NHS support after 7 months (P220, White British). Another *“found everything on my own on the internet.”* (P95, Central/East European, Stamina/Mental Health].

A few others across the ethnic groups, eligible for free NHS care, including a few white British people, turned to private care explicitly because of waiting times (*‘it feels like I cannot rely any more on the NHS.’ P219, South Asian, Food-relevant/Respiratory*), although this was not possible for maintenance of existing NHS equipment.

I was referred to a colorectal specialist for treatment but unfortunately, they put me on a very long waiting list. Luckily my husband managed to make an appointment with a private specialist where I have a colonoscopy and emergency blood test. It cost lots of money. (P191, South Asian, multimorbidity)

COVID-19 has affected follow up to review my CPAP machine and my health. It could not be assessed outside the NHS otherwise I would have looked into that. (P154, North African, Stamina)

## Discussion

As a novel contribution, this study has revealed three distinct categories of NHS treatment backlog that left participants suffering for months to years, worsening their condition and expectations of the NHS. This is particularly marked, for example, in the data that support the categories of delayed diagnosis and indefinite cessation of secondary care, where people talked about giving up or feeling suicidal as a result. It is also supported by workshop discussions of this interpretation (not reported here).

The first category considered in this paper, deferrals, occurred when patients put off help-seeking because of known NHS issues or fear of COVID-19, or where primary care diagnosis processes were frozen by healthcare providers (this latter representing 1.6 million diagnostic tests).^40^ Warner and Zaranko^8^ noted the reduction in patient help-seeking at the start of the pandemic led to a temporary fall in the elective care waiting lists; the CICADA study data show some of this reduced help-seeking was a consequence of waiting list times. The CICADA data also give concrete examples to address Warner and Zaranko’s comment that this reduced help-seeking ‘represent(s) a major puzzle’ that current NHS data cannot solve.^8^ It is estimated that during the pandemic over 10 million patients did not come forward for treatment who might otherwise have done so.^9^ Such deferrals raise concerns regarding the pandemic’s health legacy.^36^ It is unclear whether some simply will never come forward for various reasons (including worsening illness and death). Certainly, while in 2023 there had been an 11% increase in diagnostic testing over 2019 levels, and the number of people joining the waiting lists had risen to similar levels to those prepandemic, this was not to the levels expected if those deferring during the pandemic were coming forward.^8 9 37^ Any that do so will be entering the care system at a later date than they would have, with no prospect of care for months to years still, or requiring more costly or complex support that could have been prevented. New diagnostic hubs, while helpful, lack staffing and some equipment.^2 40^

My second category comprised waiting lists. The first type is known as the ‘elective care’ waiting list, the ‘RTT’ waiting list, or the ‘incomplete care pathway’. The UK Government’s elective recovery taskforce (ERT), set up in December 2022, was intended to help NHS England tackle this, for example, by increasing the NHS workforce and use of the independent sector. This followed the Elective Backlog Recovery Plan, published in February 2022, in which NHS England set several elective waiting list targets, including committing to 9 million more tests and checks by 2025 and investment in an expanded network of community diagnostic centres.^41^ However, in January 2024, almost 7.58 million were on a waiting list for hospital treatment in England^2 8^; this included 6.3 million unique patients, indicating that some were waiting for more than one procedure. At this time, 62.3% of patients nationally had been treated within 62 days of referral, failing to attain the 85% standard (which has not been reached since September 2015).^8^ During the pandemic, elective care waiting lists became so long that some GPs delayed, cancelled or refused referrals into secondary care waiting lists. Since this is a direct consequence of the long lists, I have included this action as a waiting list (for referral). Starmer’s plans particularly address this issue. They tackle the problem in a different way to systems in other countries, as noted by Central and East European participants; instead of patients being able to self-refer to consultants, bypassing the GP, Starmer’s plan bypasses the consultant initially.

The third category is called delays, which occurred when existing treatment, monitoring plans or social care were reduced or cancelled (whereas those on waiting lists had not had such plans drawn up). This was often poorly managed according to CICADA participants and left people in limbo. It is worth noting that, as table 3 shows, the CICADA data support differentiation between apparent cancellations and delays that were perceived as temporary, and the BMA does not,^36^ whereas the BMA makes a similar distinction for diagnostic tests,^36^ which seems important, though the CICADA data do not show this. This indicates where further research might be useful. Perhaps the three typologies, mine and those derived from the BMA and IFS publications,^36 37^ should be combined.

Participants on waiting lists and those experiencing the cancellation of planned care felt in limbo, particularly in later interviews, suggesting that the longer the delay, the more likely this feeling was to develop, or else that (resumption of) services had become less certain as the pandemic wore on. Existing patients given a revised date were initially less likely to feel in limbo, a novel finding showing how a simple action can reduce patient stress and potentially improve clinical relationships. Several participants reported being sufficiently empowered to take further action to get NHS care.

How do the CICADA findings relate to the different solutions suggested in the various UK election party manifestos of 2024? The victorious Labour party said it aims within 5 years for compliance with NHS waiting time performance standards. To achieve this, Labour plans to incentivise staff to work ‘out-of-hours’, and pool resources across hospitals in the same area so that they share waiting lists, as well as drawing on spare capacity in the private (independent) sector. These plans are in addition to Starmer’s January 2025 announcement. I have previously explored how NHS secondary care staff already do a lot of work on ‘goodwill’,^3^ and more recent reports discuss the resultant burn-out and attrition, so the success of this is perhaps questionable.^24^,^6 37^ However, by removing one consultant visit from the diagnostic process, the burden on staff may reduce over time. This could also have a knock-on effect on the other backlog categories I have considered here; for example, it may encourage more patients to come forward for diagnosis (deferrals type B). Labour also says it will invest in new hospitals to tackle the poor state of NHS estates, coupled with fundamental reform. It plans to develop an NHS innovation and adoption strategy in England, modelled on Covid infection control and vaccine development strategies.^42^ This it says will result in an extra 2 million NHS operations, scans and appointments every year. Labour also plans to modernise the NHS with investment, for example in state-of-the-art diagnostic equipment. These strategies should cut directly across the different categories of backlog described in this paper if done well. However, a further plan is needed to explicitly reach those people who did not seek help during the pandemic and may still not have come forward (deferrals B) even if they may be encouraged indirectly as noted above. The Liberal Democrats instead might have particularly impacted this group, focusing on improved access to GPs. The exiting Conservatives promised to build 40 new hospitals and 50 new community diagnostic centres by 2030, thus, like Labour, potentially impacting across the different categories, if this was achievable, especially in the light of staff attrition. The other significant party, the Reform Party, pledged to inject the most money of any party into the NHS and introduce basic rate tax relief on private healthcare, and by such measures reduce NHS waiting lists to zero within 2 years. However, this seemed an unrealistic promise,^37^ and there was no clear plan as to how this would address the different types of backlog.

In terms of minoritisation and intersectionality considerations, CICADA data indicate that a lack of interpreters could lead to delays, but there was no clear evidence to show that Asian or younger black people had a markedly different experience because of their ethnicity specifically, in contrast to the Nuffield report which was based on a survey of larger numbers.^25^ While more South Asian people reported giving up help-seeking, there were twice as many South Asian participants as any other group and an equivalent number from South Asia proactively complained to providers. White British and Central and East European participants seemed more empowered than others in navigating the systems. Overall, impairment type was considered a more significant factor affecting backlogs than ethnicity, though unexplained subjectivities in decision-makers were noted in terms of who, rather than which condition, should be prioritised. Participants with mental health issues and neurodivergent participants were the most likely to feel neglected and left in limbo. Level of deprivation (or socioeconomic status) affected social care access more than healthcare access, although it is known to be linked to worse health in the first place, both as a causal factor and as a consequence of structural discrimination. The King’s Fund found that waiting lists rose fastest in deprived areas in 2020–2021 but that this plateaued in 2021–2022 at the time that the CICADA study was ending.^21^ This, as the King’s Fund suggested, might have various explanations. The NHS Strategy Unit found that patients in the most deprived areas are more likely to receive an initial diagnosis from a GP but are less likely to receive secondary care treatment.^43^ It is not clear whether Starmer’s plans can address this. The King’s Fund suggested this showed a mismatch between a lower supply of elective care and a higher demand in more deprived areas.^21^ CICADA data suggest additional factors, such as patients simply dropping out and turning to alternative sources of support, which CICADA data not reported here indicate may be more likely in, for example, diasporic communities. Participants who resorted to private healthcare did so because of desperation and often could not afford this. CICADA data support another King’s Fund hypothesis, that processes for patients from more deprived areas are slowing down the treatment pathway and that closely linked external factors should be considered, such as funding allocations and workforce availability in different areas. In concordance, CICADA participants noted access difficulties of various kinds, including but not limited to language and a lack of knowledge of processes, as well as reporting a postcode lottery.

Warner and Zaranko determined that the elective waiting list in December 2023 was 113% higher in the East of England than in January 2020, but 71% higher in the North East & Yorkshire.^8^ Although the CICADA study compared different regions, I was not able to demonstrate this particular inequity. However, like these authors, I did find a difference between clinical specialties. For example, Warner and Zaranko reported that in December 2023, the waiting list for general internal medicine was 2% lesser, and for gynaecology 109% greater than in January 2020.^8^ Similarly, I found participants specified the longest waiting times for endometriosis (1 year) and mental healthcare (up to 2 years). These specialties should be a particular focus of the government. Endometriosis, which affects 1.5 million women in the UK, takes an average of almost 9–10 years to be diagnosed in the UK, a much longer time than for many other conditions that have shorter waiting lists, and an increase of 10 months from 2020.^43^ So the prolonged gynaecology treatment waiting lists add insult to injury and compromise the physical and mental health of these women. The damage caused by the psychological impacts of the pandemic has been well documented.^38 39 44^

### Strengths and limitations

This is, so far as I am aware, the most in-depth study of the lived experiences of people from disabled people from minoritised ethnic groups, in the context of their health and social care. The team successfully used community coresearchers to increase reach and include undocumented migrants and others with precarious status. The team recruited across a wide range of disabilities and ethnic groups, and the analysis drew on interviews with 271 people.

Nonetheless, there were challenges. Though many interviews were face-to-face, a majority were online and thus may have excluded some older and more disabled participants. There were formal translators/interpreters in only two of the six sites, though informal interpreters and lay coresearchers supported participants elsewhere, and consent was fully informed across all sites. The study sampling and analysis focused on England, though there are limited data from Wales and Scotland showing similar accounts. While data from 271 participants were considered for context for the analysis reported here, specific details came from only 73 of these. However, this is still a significant number, larger than for most qualitative studies, and the remaining participants will generally not have been affected because they did not require ongoing care, for example, those self-managing their condition successfully, or who had reached a point where no further treatment was helpful. The qualitative data have stronger representation by South Asian participants than others, though this was corrected for in analyses. We were able to include some longitudinal analysis as we collected interview data from 1 July 2021 to 15 September 2022, but as data collection was continuous over this time, we could not easily divide the analysis into different specific stages or regions, and we drew on recall of experiences during 2020 including the first lockdown.

## Conclusions

While enhanced levels of treatment and diagnosis, as proposed by the UK government, cut across all types of NHS care backlogs, different types of backlog require different additional considerations. Better access to general practice may encourage those people to seek healthcare help who ceased doing so during the pandemic. Referral processes, currently being reformed, need consideration of inequities and may increase rather than remove GP bottlenecks. Patients on long waiting lists or who have appointments cancelled should be kept in the loop about when their next appointment is likely, without promising unrealistic targets. Patients deserve greater transparency regarding the prioritisation of conditions to deal with waiting lists and delays. The government needs to ensure neglected specialties such as gynaecology and those currently overloaded such as mental healthcare are particularly supported over the next few years. This may include supporting alternative services rather than the increasing privatisation of healthcare. Work is needed to restore patient confidence, with a need for appropriate information and realistic promises that will encourage those who stopped help-seeking to come forward and prevent further deterioration in their condition. The issues of waiting lists for interpreters and diagnostic, monitoring and review delays for relapsing remitting conditions deserve appropriate attention. Not least, the CICADA data can be used to inform the development of plans for future pandemics and healthcare system crises.

## Supplementary material

10.1136/bmjopen-2024-091182 online supplemental file 1

10.1136/bmjopen-2024-091182 online supplemental file 2

## Data Availability

Data are available on reasonable request.
