# Supplementary material for: Towards a better understanding of NHS secondary and social care backlogs: qualitative perspectives on waiting lists, deferrals and delays by disabled people from minoritised ethnic groups
Source: BMJ Open. 2025 Apr 14;15(4):e091182. doi: 10.1136/bmjopen-2024-091182 (PMC11997839; doi:10.1136/bmjopen-2024-091182)
Supplement: online supplemental file 1 [file bmjopen-15-4-s001.docx]

**CICADA-ME (Coronavirus Intersectionalities: Chronic Conditions and Disabilities and Migrants/Ethnic minoritisation)**

**Topic Guide:**

Note: We want to know about the current situation for people, but also about how the pandemic has been for them. So although you should focus on the present, you should also probe throughout on changes over the last year to now (especially for those for whom life feels pretty much normal now).

Below we give the topic summary or aim and then some possible questions (some questions are different ways of finding out about the same thing, so you do not have to use them all, choose which style works best for you and the participant, or use your own versions). Also, people often answer one question with a response that means you do not have to ask some other questions because they have covered them in their one answer!

Throughout we are interested in the impact of their health condition or migrant/minoritized status **so you could ask throughout or just at the end** “Is your ethnic or racial background/visa status or health condition more relevant to this? (Please explain)”

1. **Intersectionalities. Aim:** To describe/situate circumstances, in relation to the intersections of chronic condition/disability and ethnicity/citizenship state, in context of UK pandemic.

*Possible questions*

How are you? Can you tell me a bit about yourself? Describe your circumstances, in relation to your ethnic or racial background and the pandemic. How have you managed the pandemic? What has worked well or less well, in your life during the pandemic? How could your experiences be improved?”

How much of this do you think is due to the pandemic itself? (would you have similar experiences even without the pandemic?)

1. **Behavioural responses. Aim:** To understand how people have managed COVID risk-reduction measures (including vaccination), as individuals and in contacts with their formal/informal support and care networks (e.g. friends, family, community, health/social care). To understand the context of peoples’ lives, what responses are feasible or acceptable to them, and effects on their networks – in relation to support and care.

*Possible questions*

What changes have you made because of worry about COVID infection? Can you describe what was hard for you to change or do? (Why?)

What can you tell me about covid vaccination?

1. **Access: Aim:** An understanding of access to resources, formal/informal support and care, including **digital transformation**, service innovations. A focus on healthcare and social services.

*Possible questions*

What support and resources have you been able to access, which have supported your health and mood in the pandemic?

(Probe: remote health appointments, use of social media/Zoom etc to socialise)

Would you have similar experiences even without the pandemic? (Please explain)

1. **Social network**: **Aim:** To get descriptions of the person’s formal/informal support and care networks) (which they might have mentioned already but this is to get a complete picture).

*Possible questions*

Can you describe the support and care you receive, formally and informally? What are your support networks? Where do you find the care you need?

Would these be similar even without the pandemic?

What is your job here? What was it back home? What social class do you perceive yourself as being in?

1. **Coping: Aim:** To learn about coping strategies and physical and mental health consequences of the pandemic, why they arose and how issues can be mitigated.

*Possible questions*

What impact has the pandemic had on your physical and mental health? How have you coped with this impact? How have you dealt with it?

Probe for whether these were existing conditions and for long covid.

1. **Local/regional differences. Aim:** To determine if where you live affects responses (for example there may be variations in available formal support)

*Possible questions*

How does your local area make an impact on your healthcare, your life, your wellbeing? What has worked well and less well for health and wellbeing outcomes, where you live? Is it different, to where you lived before (in a positive or negative way)? And how has the pandemic affected it?

1. **The future. Aim:** So we can recommend new policy that is accessible to people with chronic disabilities and minoritised ethnic groups.

*Possible questions*

How would you change the policies which exist, in relation to your disability or ethnicity, to improve your life? What changes do you think are needed? How has the pandemic suggested any of these to you? How has the pandemic affected the way you think about these?
