# Supplementary material for: Towards a better understanding of NHS secondary and social care backlogs: qualitative perspectives on waiting lists, deferrals and delays by disabled people from minoritised ethnic groups
Source: BMJ Open. 2025 Apr 14;15(4):e091182. doi: 10.1136/bmjopen-2024-091182 (PMC11997839; doi:10.1136/bmjopen-2024-091182)
Supplement: online supplemental file 2 [file bmjopen-15-4-s002.docx]

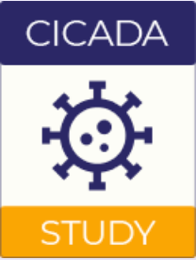
Topic guide for follow up interview and discussion groups

The overall objective of the second interview or discussion is to understand how things have changed for the participants since the first interview.

**Opening:**

Introduce the objective of this discussion, which is to understand how things have changed for you, since the last interview, or in 2022, which might be easier for people to grasp.

How are you now since the pandemic measures have lessened?

Looking back on your pandemic experiences what do you think have been the most lasting changes for you that are still continuing?

(This could be health impact or it could be changes in the way services are provided or the family support…. Anything really)

**(intersectionalities)**

How does your disability/condition currently influence your experience?

Do you think your ethnicity affects these experiences?

**(behavioural responses)**

What things, if any are you still taking to protect yourself from COVID?

What do you think about how things are opening up now?

Did you get any vaccinations? If so, did you get the boosters and how many? What factors helped you make your decision to get the vaccine or not get it?

Are there things that you still have to do because of the pandemic that you find difficult to adjust to?

**(Access to services, resources, formal and informal support)**

How are you currently finding access to healthcare (GP, hospital appointments, medications etc)?

How do you think your disability/condition or ethnicity/visa status relates to this?

What other services and resources are helping you now? (ask e.g. healthcare and community support - community centres, informal resources on line, formal resources on line, use of food banks, family doctors, leisure activities, any other activities (volunteering, charities etc)

**(Immediate people in your network)**

How have your relationships with friends and family and neighbours changed as the pandemic is improving?

What help do they give you?

Are there things you miss about the these relationships during the pandemic? Such as?

Are there things that have improved? Such as?

**(Coping)**

Overall, how do you think you are coping now and how has this been affected by the pandemic?

*Probe:*

How is your mental health overall?

How is your physical health over all?

What do you think is the most helpful thing you do or experience that helps you cope?

What would make things easier for you now and would help you cope better?

**(Local area/future)**

Looking back what services or people in your local area were most helpful to you during the pandemic?

How has that changed now?

What changes either locally or nationally do you think would help you cope better?

**(Close)**

Is there anything else you would like to tell me about how you are coping now or how things have changed for you compared to during the pandemic?
