# Supplementary material for: Towards a better understanding of NHS secondary and social care backlogs: qualitative perspectives on waiting lists, deferrals and delays by disabled people from minoritised ethnic groups
Source: BMJ Open. 2025 Apr 14;15(4):e091182. doi: 10.1136/bmjopen-2024-091182 (PMC11997839; doi:10.1136/bmjopen-2024-091182)
Supplement: Supplementary file 3 [file bmjopen-2024-091182.reviewer_comments.pdf]

PEER REVIEW HISTORY

BMJ Open publishes all reviews undertaken for accepted manuscripts. Reviewers are asked to complete a checklist review form and are provided with free text boxes to elaborate on their assessment. These free text comments are reproduced below.

ARTICLE DETAILS

Title (Provisional)

Towards a better understanding of NHS secondary and social care backlogs: qualitative perspectives on waiting lists, deferrals and delays by disabled people from minoritised ethnic groups.

Authors

Rivas, Carol

VERSION 1 - REVIEW

**Reviewer**	**1**
**Name**	**Kaneko, Makoto**
**Affiliation**	**Yokohama City University, Primary Care Research Unit, Graduate School of Health Data Science**
**Date**	**28-Aug-2024**
**COI**	**none**

To the authors

Thank you for your great work.

I enjoyed reading the article.

My comments below are based on the COREQ checklist for qualitative study (https://academic.oup.com/intqhc/article/19/6/349/1791966)

and I hope these comments improve your article.

Major concerns

Introduction

#1. Please state why the authors focused on “people from minoritised ethnic groups who had disabling impairments and chronic conditions” briefly for readers, even if the rationale was stated in their previous report.

Methods

#2. Did the authors use an interview guide or prompt? If so, please share the interview guide.

#3. Were field notes made during and/or after the interview or focus group? (COREQ domain 2)

#4. Please describe the number of non-participants. How many people refused to participate or dropped out? Reasons? (COREQ domain 2)

Discussion

#5. The authors mentioned British political parties and policies, which might seem abrupt to non-British readers, so they should add a little more explanation of the British political system and situation.

Minor

#1. There are two "Table 1" in the manuscript:

-Table 1: Quota sampling combinations, initially aiming for 5 per cell, and then 7 once top-up funds were received in spring 2021

and

-Table 1: Public participant demographics

Please rename them.

#2. The authors used abbreviations such as "MP", "GPs", and "NIHR" in the abstract and manuscript. Please spell out them the first time they appear.

#3. The authors used both "we" and "I", "our" and "my" in the manuscript. I think it would be better to unify the terminology.

**Reviewer**	**2**
**Name**	**Merrick, Hannah**
**Affiliation**	**Newcastle University Population Health Sciences Institute**
**Date**	**23-Oct-2024**
**COI**	**None**

This paper aimed to understand perspectives on waiting lists, deferrals and delays as a result of the pandemic by disabled people from minoritised ethnic groups. This is an important and relevant topic following the changes to services during the COVID-19 pandemic and the impact these had, particularly in disabled people. The study collected large amounts of data and has used excellent participatory methods.

The are a number of areas I think need to be addressed to make the paper stronger and to be able to understand the methods and key findings.

Introduction:

The introduction has a lot of focus on policy and current wait times in the NHS. However, on balance there needs to be more context about how service change during the pandemic has exacerbated current waitlists and the experience of delays during the different stages of the pandemic. Given the focus of the paper is also on disabled people and minoritised ethnic groups I would also expect to see some literature referring specifically to their experience of the pandemic and why increased waitlists are specifically impacting these groups compared to the general population.

The above means that the aims of the study in the final paragraph of the introductions doesn't seem to follow and we are suddenly being introduced to the experience of minoritised ethnic groups who had disabling impairments.

I think it would be helpful to spell out what the CICADA acronym stands for to help the reader understand the study focus.

Methods:

I think the methods would benefit from some subheadings to help guide the reader and add some clarity to the methods used for the overall study and what relates specifically to this paper.

Sample: I assume from Table 2 the sample was all adults aged 18+ but this is not stated in the methods. The methods in the abstract talk about a white comparator group, but the group are not described as such in the main methods. There also does not seem to be any reference to comparisons of white and minoritised ethnic groups in the results or the discussion.

What is meant by 'key informant' interviews. Who are the key informants?

It is not clear from the methods currently how participants volunteered to take part in the study. The results section mentions pre-screening but there is no mention of this process in the methods. Did participants register an interest? If so, how did they do this?

Interviews: What was the purpose of the repeat interviews? These are mentioned with the workshops but from my understanding of what is written the workshops were more about data checking and confirming interpretations. Is that correct?

When were the interviews carried out? This is important context that needs to be referred to, as the different restrictions in place between March 2020 and into 2022 will have impacted how waitlists and referrals could be handled and the experiences had.

The discussion mentions that interviews had to be done online in the limitations. This is not described in the methods. Were any done face-to-face? Was video call used or telephone?

The September workshop describes face-to-face and online participants. Was this a hybrid workshop or were separate workshops held for in person and online.

It needs to be made clearer how the data in this study was taken from the interviews from the main study. What was the focus of the interviews conducted? Was data about waitlists taken from all 271 interviews for the analysis?

Analysis: The analysis states that framework analysis was used with deductively determined themes. What informed these themes? How were they deduced? You mention inter-rate reliability, but it is not clear how many people coded the interviews or how disagreements between raters were resolved.

Results:

With the numbers that attended the workshops, did some people attend both the May and September workshop? Is there a reason why the first one was attended more than the second? Did 104 people attend one workshop in May? That may need further description of how this was managed in the methods (e.g were there breakout groups?). How were people chosen to be invited to the workshop?

Table 2 is mislabelled as Table 1 in its caption.

The results are under the sub-heading “Thematic analysis”, yet you used framework analysis.

The results section of the abstract is says “Disability type had more effect on

experiences than ethnicity, though interpreter waiting lists and referral models from other countries

were mentioned.” However, I do not see evidence or discussion of this in the results section. The objectives in the abstract talk about the impact of the backlogs but I do not think this comes through in the results. The results are very focussed on the type of waits and delay rather than how they impacted disabled people. I think the results of this paper would be made stronger by including more about the impact and drawing out the comparisons mentioned in the abstract.

On page 9, at the end of the first paragraph under the heading “waiting for referrals (type B), it is not clear to me if some of the statements are directly related to what interviewees have said or what is being drawn from the wider literature? For example, the “attribution to loneliness, social isolation and stress”.

Discussion:

The second part of the opening sentence to the discussion is not supported by the current results – “left people suffering for months to years, worsening their conditions and expectations of the NHS”.

As with the introduction, I think the discussion needs more focus on the groups you have sampled for this study. What do the results mean for disabled people, and particularly for minoritised ethnic groups who have a disabling impairment?

The strengths and limitations section needs more consideration to clearly outline the limitations of the study in full.

VERSION 1 - AUTHOR RESPONSE

Comments to Author	Response	Text change
**Reviewer: 1**		
Thank you for your great work.<br>I enjoyed reading the article.	Thank you	
Introduction<br>#1. Please state why the authors focused on "people from minoritised ethnic groups who had disabling impairments and chronic conditions" briefly for readers, even if the rationale was stated in their previous report.	Added on p 5:<br><br>This was originally in the methods where I discussed inclusion criteria and so is now deleted from there.	The focus on these groups was important. Data had emerged that showed the pandemic was widening pre-existing inequities in health and social care access and use for these groups.[17-20] A 2022 report had described deprivation as being a key marker of inequities in waiting lists specifically. Though it did not link this to ethnicity and disability, people from minoritised groups are more likely to be deprived because of structural discrimination. Therefore, my particular interest was in whether and how disability and ethnicity, along with citizenship status, intersected[21] to compound inequities because of structural discrimination.
#2. Did the authors use an interview guide or prompt? If so, please share the interview guide.	Thank you, we had mentioned them for the workshops but not the interviews, amended on P 7 and added as suppl material.	All interviews and workshops were structured using topic guides. These explored the different aspects of daily living during the study period, but the main focus was on health and social care experiences; we

		began with open questions followed by probes.
#3. Were field notes made during and/or after the interview or focus group? (COREQ domain 2)	Added on p7	Interviewers and facilitators made field notes and summaries; this helped the field researchers to gel as a team and reflect together on their cultural understandings and cultural humility.[29] It also aided quota sampling checks.
Methods<br><br>#4. Please describe the number of non-participants. How many people refused to participate or dropped out? Reasons? (COREQ domain 2)	This was convenience sampling. No-one dropped out or refused. Added p 7<br><br>Also a section in the sampling methods	**P7**<br>Interviews lasted 25-90 minutes, workshops 2 hours. Altogether 80 of the total 274 interviews were undertaken by partners and community co-researchers. No-one dropped out.<br>**Sampling bit:**<br>The original target for the qualitative work was 210 semi-structured interviews, two workshops with interviewees five and ten months later and up to 15 key informant interviews. Mixed stakeholder co-create workshops to develop rapid-impact solutions to issues, also undertaken, are reported in a separate paper. Participants were recruited using posters, adverts, snowballing and invitations sent out via the study's various networks, partners, community co-researchers and Clinical Research Networks. Convenience sampling and inclusive principles meant anyone who responded to these by showing an interest to take part did so unless screening talks pre-interview made it clear they did not fit the inclusion criteria; only six were gently told they were not eligible. One-third of interviews were undertaken face to face and 30 by telephone; the remainder were completed using Teams or Zoom. One was

		by email because the participant had difficulty talking due to an intellectual disability. All participants received £20 per interview.
Discussion<br>#5. The authors mentioned British political parties and policies, which might seem abrupt to non-British readers, so they should add a little more explanation of the British political system and situation.	I have tried to balance this with the need to keep to the word count and to not deviate too much from the main thrust. The first sentences in the paper now.	In July 2024 there was a general election in the UK; a UK Prime Minister's term of office lasts a maximum of five years. National Health Service (NHS) waiting times were a key consideration in the different party manifestos at this time, reflecting public concerns.
#1. There are two "Table 1" in the manuscript:<br>-Table 1: Quota sampling combinations, initially aiming for 5 per cell, and then 7 once top-up funds were received in spring 2021<br>and<br>-Table 1: Public participant demographics<br>Please rename them.	Corrected.	
#2. The authors used abbreviations such as "MP", "GPs", and "NIHR" in the abstract and manuscript. Please spell out them the first time they appear.	Done	
#3. The authors used both "we" and "I", "our" and "my" in the manuscript. I think it would be better to unify the terminology.	Amended, sometimes by using 3rd person	
**Reviewer: 2**		
The study collected large amounts of data and has used excellent participatory methods.	Thank you	
The introduction has a lot of focus on policy and current wait times in the NHS. However, on balance there needs to be more context about how service change during the pandemic has exacerbated current waitlists and the experience of delays	I absolutely agree, on re-reading the introduction. However, the data are patchy and insufficient regarding the different stages of the pandemic from the perspective of this paper, though I have added some text.	**Added to intro:**<br><br>These various pandemic impacts all led to patients becoming first sympathetic to NHS staff efforts and then increasingly disgruntled with what was perceived as a lack

during the different stages of the pandemic. Given the focus of the paper is also on disabled people and minoritised ethnic groups I would also expect to see some literature referring specifically to their experience of the pandemic and why increased waitlists are specifically impacting these groups compared to the general population.	Concerning our groups of interest, there really is almost nothing in the literature (and we did a scoping review) and certainly even less that is of a quality that would lead to my using it (eg very small studies not looking at this as a primary question). However, since I wrote this paper, a report has been published by the Nuffield Trust that gives a very small amount of the information you suggest, and I have been able to include it as per the next column, along with some existing survey data, and your points are carried through into the discussion. I was also able to add Starmer's new plans announced January 2025!<br><br>I have also deleted some of the introduction to get more of the balance that you suggest and to try to keep to the word count.	of caring as well as care. It was in this context that I developed the CICADA-ME study (Coronavirus Chronic Conditions and Disabilities Awareness Study-Migrants and Ethnic groups; hereafter CICADA), which aimed to explore the pandemic experiences of people from minoritised ethnic groups who had disabling impairments and chronic conditions. The focus on these groups was important. Data had emerged that showed the pandemic was widening pre-existing inequities in health and social care access and use for these groups.[17-20] A 2022 report had described deprivation as being a key marker of inequities in waiting lists specifically. Though it did not link this to ethnicity and disability, people from minoritised groups are more likely to be deprived because of structural discrimination. Therefore, my particular interest was in whether and how disability and ethnicity, along with citizenship status, intersected[21] to compound inequities because of structural discrimination.<br><br>In this paper I consider what participants said about the waiting lists and diagnostic delays that were subsequently a focus in the 2024 election party manifestos. The CICADA data remain topical. As of 2024, the NHS has still not returned to even over-stretched pre-pandemic levels of care. Ambulance responses have only slightly improved.[7] In

		January 2024, a daily average of 5,600 patients waited longer than 12 hours in Accident and Emergency (A&E).[8] The waiting list for hospital treatment following a GP referral reached a record of nearly 7.8 million in September 2023.[8,9] While England's waiting lists began to fall in 2024, the Institute for Fiscal Studies (IFS) has predicted, from current trends, that it will take years to reach pre-COVID pandemic levels.[8] In January 2025,the new UK Prime Minister, Keir Starmer, introduced plans to speed up this recovery.[22] Patients will be able to get a direct referral from their GP for diagnostic and other tests and scans, replacing the previous requirement for patients to see both a GP and then a consultant first, and with many being offered a follow-up consultation on the same day as their scans or tests. Routine surgery such as hip and knee replacements will also be protected from cancellations and delays caused by seasonal or unexpected increased demands on the NHS more generally. However, there was no mention in these plans of ways to address inequitable service provisions. In 2022, the Nuffield Trust had reported that elective care had decreased disproportionately for Asian people in the UK.[23] In October 2024 they found that people from the most deprived areas are much more likely to still be on waiting lists for elective care, that young Black people experience longer waits in A&E, and that there

		are particularly amplified waiting lists for gynaecological care.[24] They have called for more research to explore this. The CICADA study goes some way to address this need.<br><br><br>**Added to discussion**<br>In terms of minoritisation and intersectionality considerations, CICADA data indicate that a lack of interpreters could lead to delays, but there was no clear evidence to show that Asian or younger Black people had a markedly different experience because of their ethnicity specifically, in contrast to the Nuffield report which was based on a survey of larger numbers.[24] While more South Asian people reported giving up help-seeking, there were twice as many South Asian participants as any other group and an equivalent number from South Asia proactively complained to providers. White British and Central and East European participants seemed more empowered than others in navigating the systems. Overall, impairment type was considered a more significant factor affecting backlogs than ethnicity, though unexplained subjectivities in decision makers was noted in terms of who, rather than which condition, should be prioritised. Participants with mental health issues and neurodivergent participants were the most likely to feel neglected and left in limbo. Level of deprivation (or socio-economic status) affected social care access more than healthcare access, although it is

		known to be linked to worse health in the first place, both as a causal factor and as the consequence of structural discrimination. The King's Fund found that waiting lists rose fastest in deprived areas in 2020-2021 but that this plateaued in 2021-2022 at the time that the CICADA study was ending.[43] This, as the King's Fund suggested, might have various explanations. The NHS Strategy Unit found that patients in the most deprived areas are more likely to receive an initial diagnosis from a GP but are less likely to receive secondary care treatment.[44] It is not clear whether Starmer's plans can address this. The King's Fund suggested this showed a mismatch between a lower supply of elective care and a higher demand in more deprived areas.[43] CICADA data suggest additional factors, such as patients simply dropping out and turning to alternative sources of support, which CICADA data not reported here indicate may be more likely in, for example, diasporic communities. Participants who resorted to private health care did so because of desperation and often could not afford this. CICADA data support another King' s Fund hypothesis, that processes for patients from more deprived areas are slowing down the treatment pathway and that closely linked external factors should be considered, such as funding allocations, and workforce availability in different areas. In concordance, CICADA participants noted access difficulties of various kinds, including but not limited to

		language and a lack of knowledge of processes, as well as reporting a postcode lottery.
The above means that the aims of the study in the final paragraph of the introductions doesn't seem to follow and we are suddenly being introduced to the experience of minoritised ethnic groups who had disabling impairments.	I believe this is now addressed by the changes shown in the row above.	
I think it would be helpful to spell out what the CICADA acronym stands for to help the reader understand the study focus.	Done at first mention	It was in this context that I developed the CICADA-ME study (Coronavirus Chronic Conditions and Disabilities Awareness Study- Migrants and Ethnic groups; hereafter CICADA,
I think the methods would benefit from some subheadings to help guide the reader and add some clarity to the methods used for the overall study and what relates specifically to this paper.	Done	
Sample: I assume from Table 2 the sample was all adults aged 18+ but this is not stated in the methods.	Added on p 6	The focus in the qualitative work was on adults aged 18+ of Arab (Middle Eastern and North African), South Asian, African, Central/East European and White British heritage with and without disability.
The methods in the abstract talk about a white comparator group, but the group are not described as such in the main methods.	Simplified in the abstract as per the cell to the right of this one	271 participants aged 18+ with a disabling chronic condition or impairment, White British and from several minoritised ethnic groups
There also does not seem to be any reference to comparisons of white and minoritised ethnic groups in the results or the discussion.	Added	See a previous response above
What is meant by 'key informant' interviews. Who are the key informants?	Clarified In the abstract and text	Abstract:<br>"key informants" of relevant others by virtue of their work<br>P 6

		up to 15 key informant (relevant professional)
It is not clear from the methods currently how participants volunteered to take part in the study. The results section mentions pre-screening but there is no mention of this process in the methods. Did participants register an interest? If so, how did they do this?	Expanded on in the methods	Participants were recruited using posters, adverts, snowballing and invitations sent out via the study's various networks, partners, community co-researchers and Clinical Research Networks. Convenience sampling and inclusive principles meant anyone who responded to these by showing an interest to take part did so unless screening talks pre-interview made it clear they did not fit the inclusion criteria; only six were gently told they were not eligible. One-third of interviews were undertaken face to face and 30 by telephone; the remainder were completed using Teams or Zoom. One was by email because the participant had difficulty talking due to an intellectual disability. All participants received £20 per interview.
Interviews: What was the purpose of the repeat interviews? These are mentioned with the workshops but from my understanding of what is written the workshops were more about data checking and confirming interpretations. Is that correct?	Yes correct, there were two repeat interviews which is now mentioned p 6	P7:<br>while for the September 2022 workshops, £40 was offered for face-to-face participation and £20 for online (including two who chose repeat interviews),
When were the interviews carried out? This is important context that needs to be referred to, as the different restrictions in place between March 2020 and into 2022 will have impacted how waitlists and referrals could be handled and the experiences had.	I do describe this longitudinal aspect in the findings. The dates were already given broadly but I have been more specific and expanded the section to address these queries. I also make a reference to this in the extra discussion paragraph already provided above.	Separate face-to-face or online workshops (none hybrid) or, if preferred, repeat interviews, were offered in May and September 2022, for interviewees who had provided contact details and completed interviews between 1st July and 20th October 2021. Following top-up funding in

		spring 2022 the target increased to 280 interviews, with recruitment and interviews continuing from 1st May-15th September 2022. Recruitment stopped at 274 due to end of the study. Two interviews could not be used because the digital consent forms were accidentally deleted and one because the interview was accidentally deleted before transcription. All remaining 271 first interviews are included in analyses and longitudinal components are described (that is, the later interview data were compared with earlier data as part of the analysis, with differences reported in this paper).
The discussion mentions that interviews had to be done online in the limitations. This is not described in the methods. Were any done face-to-face? Was video call used or telephone?	Corrected/explained as a little misleading before.	One-third of interviews were undertaken face to face and 30 by telephone; the remainder were completed using Teams or Zoom. One was by email because the participant had difficulty talking due to an intellectual disability.
The September workshop describes face-to-face and online participants. Was this a hybrid workshop or were separate workshops held for in person and online.	Added on p8	For the follow-on research workshops in May and September 2022, 134 were invited by email; 104 attended in May with three online workshops and two face to face (in London and Bradford). In September 2022, we held two London face-to-face sessions (n=22), two small online sessions with participants from the Midlands (n=11) – converted from a local face-to-face session at their request - and two individual remote interviews. Each workshop was capped at 30 and used breakout groups or tables. Reasons for non-attendance in September were mostly that

		people had returned to their jobs and normal lives and did not have the same availability.
It needs to be made clearer how the data in this study was taken from the interviews from the main study. What was the focus of the interviews conducted? Was data about waitlists taken from all 271 interviews for the analysis?	A good point -see additions in next cell	**On p6**<br><br>Interviews and workshops were structured using topic guides. These explored the different aspects of daily living during the study period, but the main focus was on health and social care experiences; we began with open questions followed by probes. The workshops also used illustrative vignettes<br><br>**In thematic analysis section of methods**<br><br>The thematic analysis showed many participants bewailed the backlogs in secondary and social care. While data from all 271 was considered for context, 73 participants provided detail on backlogs; exploration of these data revealed the usefulness of subdividing
Analysis: The analysis states that framework analysis was used with deductively determined themes. What informed these themes? How were they deduced? You mention inter-rate reliability, but it is not clear how many people coded the interviews or how disagreements between raters were resolved.	I was hoping to keep to word count by referring people to other of our references for some of these details but agree that is not ideal and amended the paragraph as shown.	The main qualitative analysis as reported on here utilised the Framework approach27 with data management in NVivo12 (QSR/Lumivero), subsequently moved into Microsoft Excel.TM Deductively determined themes (the broad topics that shaped topic guides: Intersectionalities; Behavioural responses to the pandemic; Access to resources, support, health and social care; Social networks; Mental and physical wellbeing and quality of life; Coping; Local and regional differences; Future policy suggestions) were augmented by inductive themes. Workshop validation and an inter-rater reliability exercise between three core team members

		involving coding and discussion until values of 75%+ were achieved for the key themes), and the study's participatory work more generally, ensured credibility.
With the numbers that attended the workshops, did some people attend both the May and September workshop? Is there a reason why the first one was attended more than the second? Did 104 people attend one workshop in May? That may need further description of how this was managed in the methods (e.g were there breakout groups?). How were people chosen to be invited to the workshop?	Some of this detail was already on p 8 but I have expanded on this as already noted in an earlier response.	
Table 2 is mislabelled as Table 1 in its caption.	corrected	
The results are under the sub-heading "Thematic analysis", yet you used framework analysis.	There are different views on Framework; I was integrally involved in its early development when it was known as an approach that used a form of thematic analysis. Since then, it has changed in scope and process. However, I have changed the title as the change does not conflict with this view.	
The results section of the abstract is says "Disability type had more effect on experiences than ethnicity, though interpreter waiting lists and referral models from other countries were mentioned." However, I do not see evidence or discussion of this in the results section.	I have added this in the new discussion paragraph provided earlier to draw out the way they findings show this. There is much on this in the findings but perhaps the focus on the types has obscured it so the new discussion section is I hope helpful in this regard.	
The objectives in the abstract talk about the impact of the backlogs but I do not think this comes through in the results. The results are very	I have added this in the new discussion paragraph provided earlier to draw out the way they findings show this. There is much on this in	

focussed on the type of waits and delay rather than how they impacted disabled people. I think the results of this paper would be made stronger by including more about the impact and drawing out the comparisons mentioned in the abstract.	the findings but perhaps the focus on the types has obscured it so the discussion section is I hope helpful in this regard.	
On page 9, at the end of the first paragraph under the heading "waiting for referrals (type B), it is not clear to me if some of the statements are directly related to what interviewees have said or what is being drawn from the wider literature? For example, the "attribution to loneliness, social isolation and stress".	Agree, where I do not cite immediately it comes from participants so have amended to be clearer.	This was attributed by participants to loneliness, social isolation and stress from remote work, job loss, closed schools, stay-at-home orders, closed businesses, physical distancing, adversity experiences and infection fear. Deterioration in other conditions because of poor healthcare access was also linked to worsened mental health among participants.
The second part of the opening sentence to the discussion is not supported by the current results – "left people suffering for months to years, worsening their conditions and expectations of the NHS".	This is explicitly supported by statements across the Delayed diagnosis and Indefinite cessation of secondary care sections so I have linked the statement to these in the discussion now.	As a novel contribution, this study has revealed three distinct categories of treatment backlog that left people suffering for months to years, worsening their condition and expectations of the NHS. This is particularly marked, for example, in the data that support the categories of Delayed diagnosis and Indefinite cessation of secondary care, where people talked about giving up or feeling suicidal as a result. It is also supported by workshop discussions of this interpretation (not reported here). The first category considered in this paper, deferrals,
As with the introduction, I think the discussion needs more focus on the groups you have sampled for this study. What do the results mean for disabled people, and particularly for minoritised ethnic groups	Amended as already mentioned above, with a new section in the discussion.	

who have a disabling impairment?		
The strengths and limitations section needs more consideration to clearly outline the limitations of the study in full.	I was not sure what to do about this given the word limit but have gone through reviewer comments and tried to reflect them.	Nonetheless there were challenges. Though many interviews were face to face , a majority were online and thus may have excluded some older and more disabled participants. There were formal translators/interpreters in only two of the six sites though informal interpreters and lay co-researchers supported participants elsewhere and consent was fully informed across all sites. The study sampling and analysis focused on England though there are limited data from Wales and Scotland showing similar accounts. While data from 271 participants was considered for context for the analysis reported here, specific details came from only 73 of these. However, this is still a significant number, larger than for most qualitative studies, and the remaining participants will generally not have been affected because they did not require ongoing care, for example those self-managing their condition successfully, or who had reached a point where no further treatment was helpful. The qualitative data have stronger representation by South Asian participants than others though this was corrected for in analyses. We were able to include some longitudinal analysis as we collected interview data from 1st July 2021 to 15th September 2022, but as data collection was continuous over this time we could not easily divide the analysis into different specific stages or regions, and we drew on recall of experiences during

		2020 including the first lockdown.

VERSION 2 - REVIEW

Reviewer 1

Name Kaneko, Makoto

Affiliation Yokohama City University, Primary Care Research Unit, Graduate School of Health Data Science

Date 09-Jan-2025

COI

Thank you for revising the manuscript.

Reviewer 2

Name Merrick, Hannah

Affiliation Newcastle University Population Health Sciences Institute

Date 04-Feb-2025

COI

Thank you for the opportunity to review this paper again. Thank you to the author for addressing the comments made by both reviewers which I think has made the paper clearer and stronger. There are no further comments from me, though I think the manuscript would benefit from a read through to pick up some minor grammar errors and typos.
